# Temporary atrial septal defect balloon occlusion test as a must in the elderly

**DOI:** 10.1186/s12872-023-03046-9

**Published:** 2023-01-12

**Authors:** André Alexandre, André Luz, André Dias de Frias, Raquel Baggen Santos, Bruno Brochado, Filomena Oliveira, João Silveira, Severo Torres

**Affiliations:** 1 Department of Cardiology, Centro Hospitalar Universitário do Porto (CHUPorto), Largo do Prof. Abel Salazar, 4099-001 Porto, Portugal; 2 ICBAS – School of Medicine and Biomedical Sciences, University of Porto, Porto, Portugal; 3 Cardiovascular Research Group, UMIB – Unit for Multidisciplinary Research in Biomedicine, ICBAS – School of Medicine and Biomedical Sciences, University of Porto, Porto, Portugal

**Keywords:** Atrial septal defect, Elderly, Transcatheter device closure, Temporary balloon occlusion test, Concealed left ventricular diastolic dysfunction, Case report

## Abstract

**Background:**

Atrial septal defect (ASD) can often remain asymptomatic until adulthood. It still remains unclear whether large ASD closure in senior people should be performed or not. Temporary ASD balloon occlusion test has been suggested as a tool to assess the risk of acute left ventricular heart failure post-ASD closure, and it allows to better distinguish responders from non-responders.

**Case presentation:**

An 83-year-old man with a long-standing uncorrected secundum ASD was admitted for recently decompensated right-sided heart failure. During hospitalization, this patient was studied with trans-esophageal echocardiography, cardiac magnetic resonance imaging, and right heart catheterization, showing high Qp:Qs ratio and favorable anatomical conditions for percutaneous closure. Because of patient’s increasing need for intravenous diuretics and worsening renal function, it was considered that transcatheter ASD closure could improve symptoms, hence it was performed an attempt of percutaneous closure of the ASD with a fenestrated device. Unfortunately, irrespective of ASD being hemodynamically significant, it was found a very significant increase in pulmonary capillary wedge pressure during the temporary balloon occlusion test, supporting the existence of concealed left ventricular diastolic dysfunction. As a result, it was decided to abandon the procedure and not to close the ASD.

**Conclusion:**

This clinical case illustrates the value of temporary balloon occlusion test before permanent percutaneous closure of ASD in elderly patients, regardless of left ventricular (systolic or diastolic) dysfunction.

## Background

Atrial septal defect (ASD) is the second most common congenital heart malformation diagnosed in adult life [1, 2]. The presenting symptoms are commonly palpitations and exercise intolerance [1]. If left untreated, this defect may result in right-sided heart failure, arrhythmia, or pulmonary hypertension [3]. Irrespective of symptoms, ASD closure is the treatment of choice for hemodynamically significant defects with evidence of right ventricle volume overload and in the absence of fixed pulmonary hypertension or left ventricle (LV) disease [2, 4–6]. Transcatheter device closure has become the method of choice for secundum ASD closure (rather than surgery) when conditions are favorable [3, 4]. Percutaneous closure of ASD in children and young adults is recommended, since it is a low-risk procedure with good long-term prognosis [7]. However, the benefits of this procedure in senior people are not totally clear, and few data are available about the results of this procedure in patients > 60 years of age [1, 2, 5, 7]. Temporary ASD balloon occlusion test has emerged as a tool to assess the risk of acute left ventricular (LV) heart failure post-ASD closure. Considering this, the authors present a case of an elderly patient with a long-standing uncorrected secundum ASD and right-sided heart failure, in whom it was decided to perform an attempt of transcatheter ASD closure.

## Case presentation

An 83-year-old man with history of hypertension, diabetes mellitus, seronegative rheumatoid arthritis (controlled with hydroxychloroquine), and permanent atrial fibrillation, presents an uncorrected secundum ASD with predominant left-to-right shunt, previously known since his 40s but not addressed by doctor-patient shared decision (considering patient’s good functional capacity and absence of symptoms). At the age of 83, the patient had his first hospitalization for acute heart failure. He stayed hospitalized for two weeks at Internal Medicine department, and he was discharged with partial improvement of complaints after optimization of diuretic therapy. Three months later, the patient was readmitted with decompensated right-sided heart failure. He complained of dyspnea for mild exertion (NYHA functional class III) and fatigue. Physical examination revealed congestive signs (periorbital edema, ascites, lower limb, and testicular edema), blood pressure 125/70 mmHg, heart rate 67/min, peripheral oxygen saturation 96% in room air, pulmonary auscultation with bilateral crackles in the lower 2/3 fields, and arrhythmic cardiac auscultation with fixed splitting of the second heart sound and a distinct systolic murmur in the pulmonic area.

**Fig. 1:**
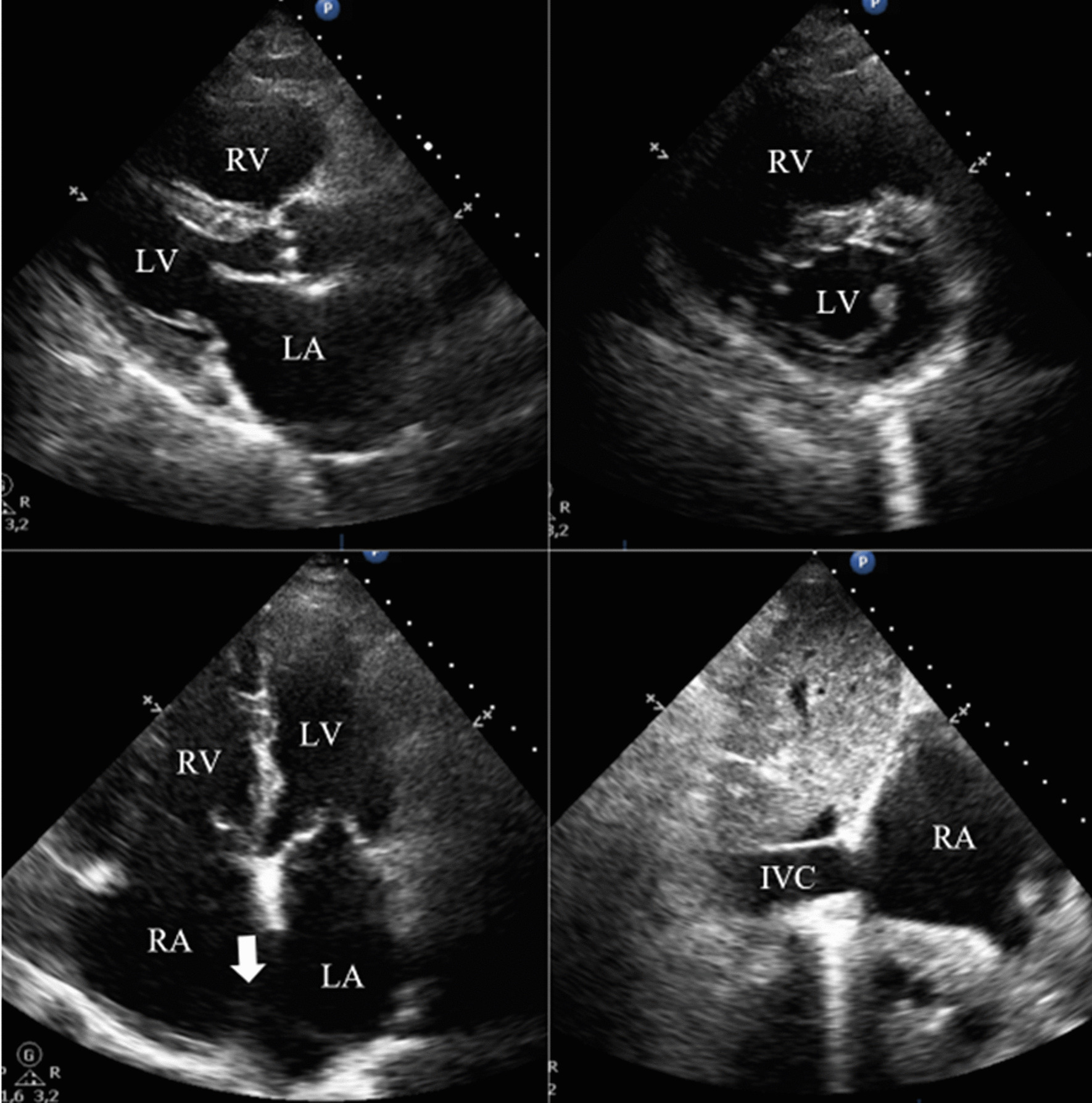
TTE imaging showing severe dilation of right cardiac chambers and RV volume overload secondary to a secundum ASD. During hospitalization, a TTE was performed demonstrating an ostium secundum ASD (white arrow) with significant left-to-right shunt (Qp:Qs = 2.6); moderate tricuspid regurgitation, estimating a PASP of 50 mmHg; severe dilation of right heart chambers and LA; dilation of IVC (27 mm) with respiratory variability < 50%; interventricular septal flattening suggesting RV volume overload; preserved global LV systolic function (+/− 63%); preserved RV systolic function; and indeterminate diastolic dysfunction. *ASD* atrial septal defect, *IVC* inferior vena cava, *LA* left atrium, *LV* left ventricle/cular, *PASP* pulmonary artery systolic pressure, *Qp:Qs* pulmonary-systemic flow ratio, *RA* right atrium, *RV* right ventricle/cular, *TTE* transthoracic echocardiography

**Fig. 2:**
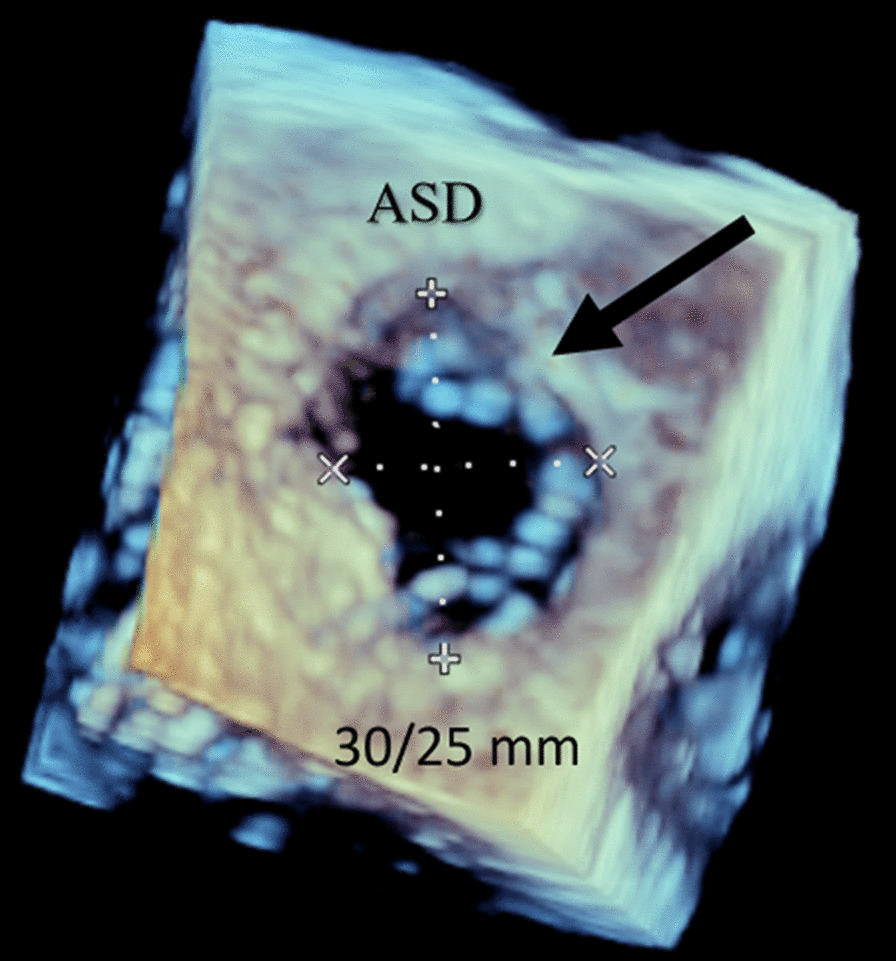
3D imaging of the secundum ASD on TEE. 3D imaging of the ASD on TEE has confirmed a secundum ASD (black arrow) with left-to-right shunt, maximum end-to-end diameter of 30/25 mm, area +/− 4.2 cm^2^, and adequate rims size (12 mm superior vena cava rim, 16 mm inferior vena cava rim, 11 mm atrioventricular valve rim, < 5 mm aortic rim). *3D* three-dimensional, *ASD* atrial septal defect, *TEE* trans-esophageal echocardiography

**Fig. 3:**
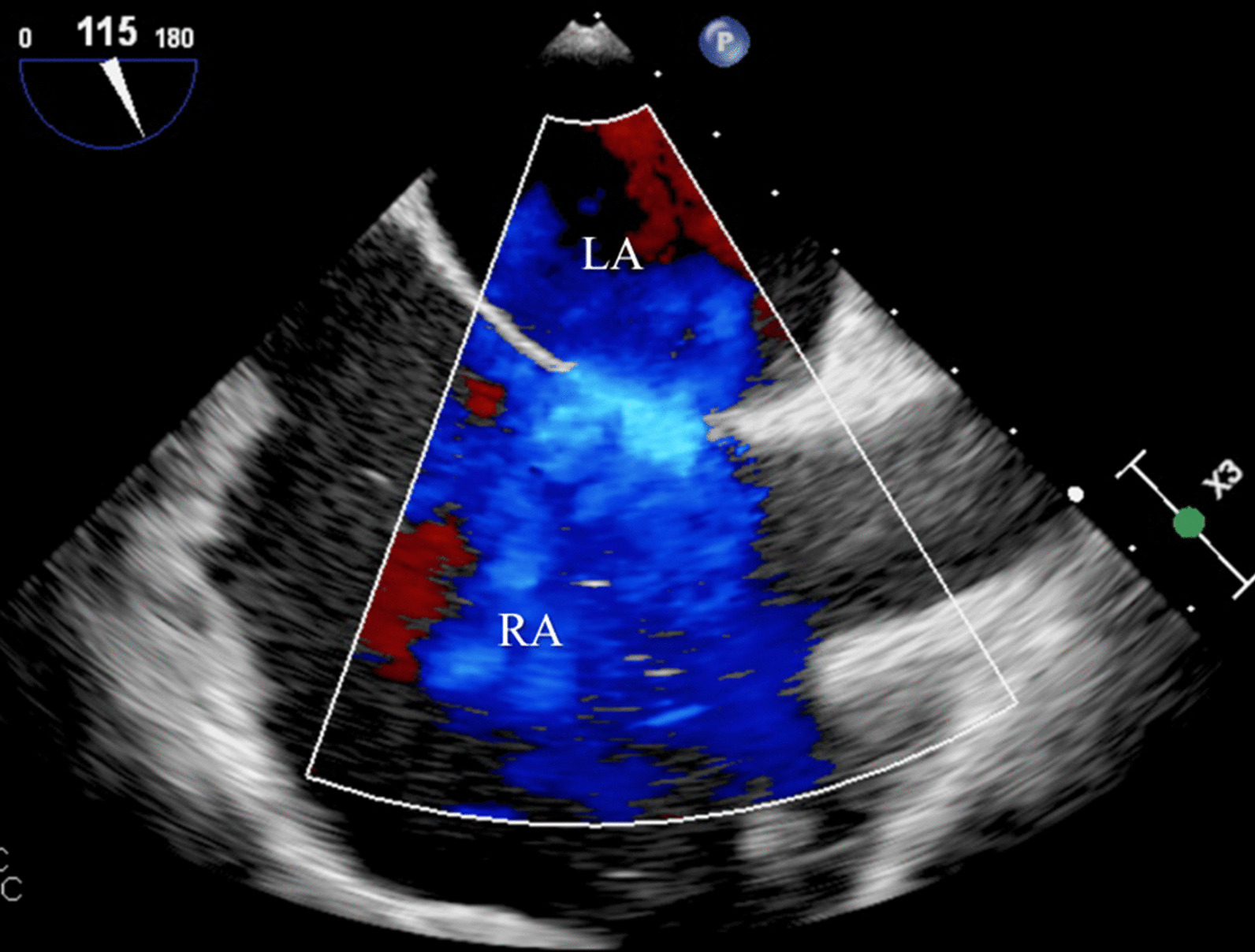
Color doppler imaging showing a secundum ASD with left-to-right shunt on TEE. *ASD* atrial septal defect, *LA* left atrium, *RA* right atrium, *TEE* trans-esophageal echocardiography

Blood analysis revealed anemia (10.4 g/dL), thrombocytopenia (87,000/µL), worsening of renal function (creatinine 2.44 mg/dL), and NT-pro-BNP 4106 pg/mL. ECG showed atrial fibrillation (50–60/min) and an incomplete right bundle branch block. Chest radiograph exhibited signs of interstitial edema and increased pulmonary vascular markings of left-to-right shunt vascularity. Transthoracic echocardiography (TTE) demonstrated (as shown in Fig. 1): an ostium secundum ASD with significant left-to-right shunt (Qp:Qs = 2.6); moderate tricuspid regurgitation, estimating a pulmonary artery systolic pressure (PASP) of 50 mmHg; severe dilation of right heart chambers and left atrium (LA); dilation of pulmonary artery and its branches; dilation of inferior vena cava (27 mm) with respiratory variability < 50%; interventricular septal flattening suggesting right ventricular (RV) volume overload; preserved global LV systolic function (left ventricular ejection fraction 63%); preserved RV systolic function (tricuspid annular plane systolic excursion 26mm; fractional area change 48%); and indeterminate diastolic dysfunction (LA volume index 53 mL/m^2^; tricuspid regurgitation velocity 3.1 m/s; septal e′ velocity 8.7 cm/s; lateral e′ velocity 11.5 cm/s; average E/e′ 9.5). To better characterize the defect, it was performed a trans-esophageal echocardiography (TEE) which has confirmed a secundum ASD with left-to-right shunt, maximum end-to-end diameter of 30/25 mm, area 4.2 cm^2^, and adequate rims size (12 mm superior vena cava rim, 16 mm inferior vena cava rim, 11 mm atrioventricular valve rim, < 5 mm aortic rim). Three-dimensional (3D) and color doppler imaging of the ASD on TEE are shown in Figs. 2 and 3, respectively. A cardiac magnetic resonance imaging was also performed, confirming echocardiographic features. Right heart catheterization features were as follows: mean pulmonary artery pressure (mPAP) = 26 mmHg; pulmonary capillary wedge pressure (PCWP) = 18 mmHg; pulmonary vascular resistance (PVR) = 1.17 Wood units; Qp:Qs ratio = 3.14; and cardiac output = 7.04 L/min by Fick method.

**Fig. 4:**
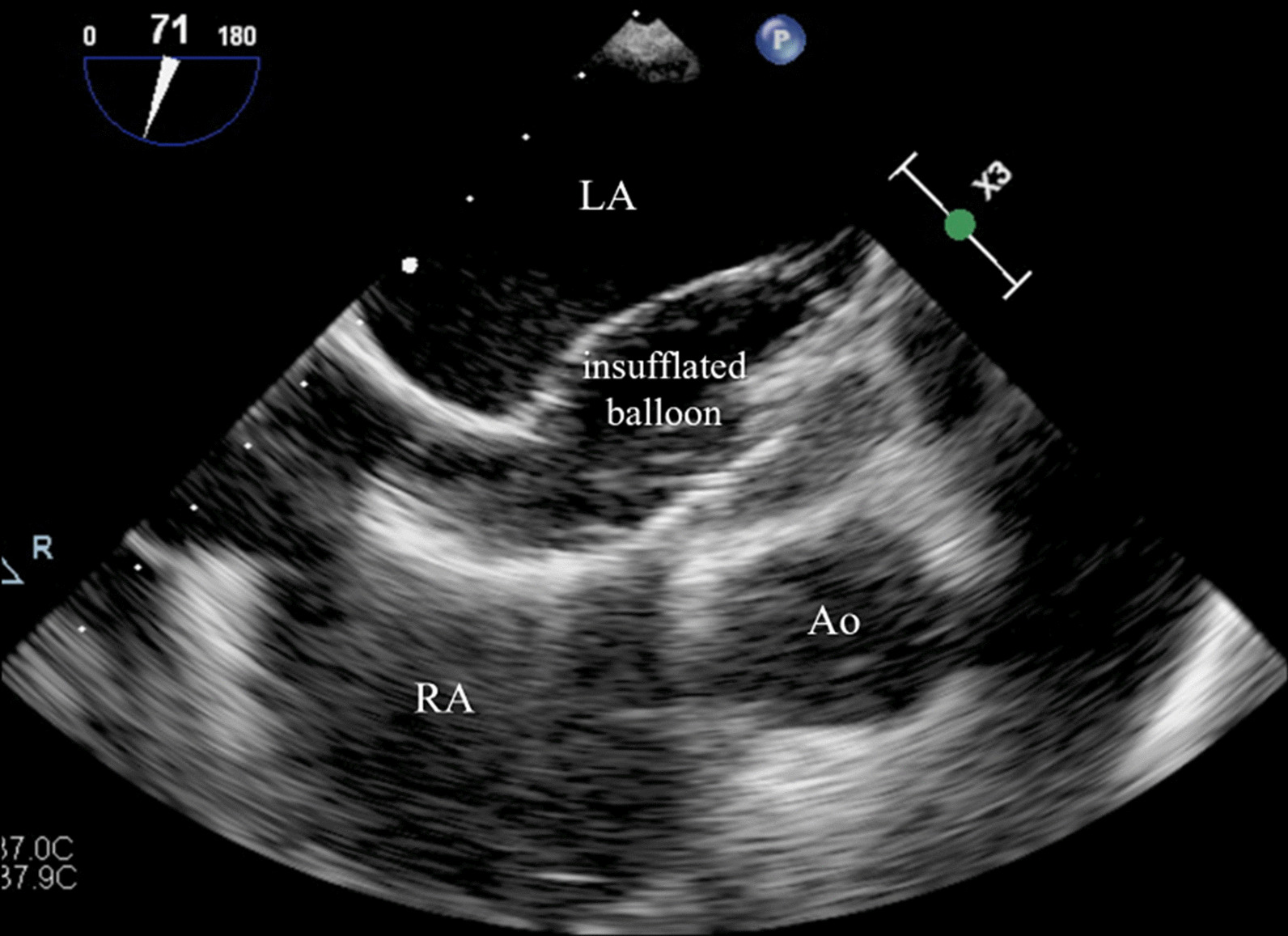
Temporary balloon occlusion test of the ASD with TEE guidance. Before permanent percutaneous ASD closure, a temporary balloon occlusion test was performed in order to check for the increase in pulmonary pressures. *Ao* aorta, *ASD* atrial septal defect, *LA* left atrium, *RA* right atrium, *TEE* trans-esophageal echocardiography

**Fig. 5:**
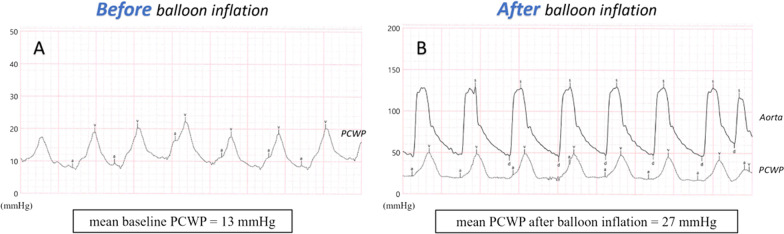
Mean pulmonary capillary wedge pressure (PCWP) measured before (**A**) and after (**B**) balloon inflation. **A** Baseline mean PCWP measured before temporary ASD balloon occlusion test was 13 mmHg. **B** Mean PCWP measured after balloon inflation was 27 mmHg. During the temporary balloon occlusion test, it was found a very significant increase in PCWP (13 mmHg → 27 mmHg) supporting the existence of concealed left ventricular diastolic dysfunction. Consequently, it was decided to abandon the procedure and not to close the ASD. *ASD* atrial septal defect, *PCWP* pulmonary capillary wedge pressure

To sum up, this is a case of an elderly patient with right-sided heart failure secondary to a long-standing uncorrected secundum ASD with high Qp:Qs ratio and favorable anatomical conditions for percutaneous closure. Although diastolic dysfunction was considered indeterminate, it may be insufficient to predict outcome in this frail population, in whom some type of diastolic dysfunction is very prevalent [8]. Given the patient’s increasing need for intravenous diuretics and worsening renal function, it was considered that transcatheter ASD closure could prevent hospitalization recurrences and provide better quality of life. Thus, an attempt of percutaneous closure of the ASD with a fenestrated device was performed under TEE guidance. During the procedure, baseline mPAP and PCWP were measured (20 mmHg and 13 mmHg, respectively), ascertaining the absence of significant pulmonary hypertension. Then, a temporary balloon occlusion test was made to check for the increase in pulmonary pressures (Fig. 4). Balloon occlusion was held for approximately 10 min. Unfortunately, a significant increase in PCWP (13 mmHg → 27 mmHg; shown in Fig. 5) was immediately found after balloon occlusion and it was sustained as long as the balloon was inflated, supporting the existence of concealed LV diastolic dysfunction unmasked by balloon testing (since the ASD may serve as an unloading pathway). Considering the risk of developing acute heart failure, it was decided to abandon the procedure and not to close the ASD. The patient was oriented to medical treatment and optimization of diuretic therapy. After proper doses of intravenous loop diuretics and sequential nephron blockade using metolazone, he was discharged with symptomatic improvement, but maintaining lower limb edema despite being medicated with high-dose diuretics. At 2-months follow-up, the patient was stable on high-dose oral diuretics, in spite of complaints of shortness of breath for mild exertion (NYHA functional class III), fatigue, and lower limb oedema. Regrettably, he died 4 months after discharge due to SARS-CoV-2 pneumonia.

## Discussion and conclusions

As described above, ASD can often remain asymptomatic until adulthood, and some patients only develop symptoms beyond the fourth decade [1, 3, 4, 6]. Nevertheless, if left untreated, these defects may result in right-sided heart failure, as showed in the present case. Hence, irrespective of symptoms, closure of ASD is the treatment of choice for hemodynamically significant defects in the absence of fixed pulmonary hypertension or LV disease, especially in younger patients [2, 4–6].

The benefits of ASD closure in senior people are less clear, and extrapolation of studies on younger patients is not appropriate in the geriatric population since hemodynamic features are significantly different between young adults and elderly [3]. First, elder patients present with comorbid conditions such as hypertension, arrhythmia, coronary artery disease, or LV diastolic dysfunction [3, 9]. Second, elder ASD patients often have inherently superior resilience, milder disease, and a balanced physiology that has allowed them to survive to an advanced age [3]. Over the past few years, an increasing number of elderly patients have been admitted for percutaneous ASD closure [1]. Some few studies have shown that transcatheter ASD closure in geriatric patients contributes to significant clinical benefit, improving symptoms, functional capacity, and ventricular remodeling [1, 2, 7, 9]. On the other hand, it has been found that a subset of elderly patients does not improve after ASD closure, and several studies suggest that the procedure can lead to the development of acute congestive heart failure due to an abrupt elevation in LV preload, especially in elderly patients with previous impaired systolic or diastolic function [2, 3, 7, 9–11]. Indeed, the ASD may serve as an unloading valve, and concealed LV diastolic dysfunction will be unmasked when the defect is closed [2]. Accordingly, if an ASD is diagnosed after the age of 60 years and symptoms are not severe, it is always a question whether the closure is advisable [1].

Considering this problem, temporary ASD balloon occlusion test has emerged as a tool to assess the risk of acute LV heart failure post-ASD closure [2, 10, 11]. Some authors suggest that if PCWP increases to a value > 20 mmHg or > 10 mmHg from the baseline value during balloon occlusion, it is expected a high risk of acute pulmonary edema and the procedure should be abandoned [3]. According to Nakagawa et al. [9], an increase > 5 mmHg from the baseline value of PCWP should be sufficient to interrupt the procedure. On the other hand, creation of a fenestration hall in the device may avoid the abrupt hemodynamic chance after the transcatheter closure of ASD [3]. Nevertheless, the optimal fenestration size has not been evaluated, data on the utility of temporary balloon occlusion test are scarce, and the experience in the elderly is still limited [9, 10].

This clinical case highlights the value of temporary balloon testing before permanent percutaneous closure of ASD in an elderly patient. In the case above, ASD closure was assumed to a high risk of developing acute LV heart failure, thus the procedure was abandoned. The significant increase in pulmonary pressures during balloon inflation (despite baseline average E/e′ of 9.5 on TTE) supports the existence of concealed LV diastolic dysfunction unmasked by temporary balloon occlusion test. Although a fluid challenge has been described in these scenarios, we decided not to use it because of the already observed excessive increase in left-sided filling pressure after transient balloon occlusion alone [12].

In conclusion, temporary balloon occlusion test is decisive in identifying patients with concealed LV diastolic dysfunction and acquires greater importance in the elderly. Performing balloon testing before permanent percutaneous closure of ASD may be considered in the majority of patients older than 65 years of age, regardless of left ventricular (systolic or diastolic) dysfunction.

## Data Availability

The data that support the findings of this study are available from the corresponding author upon reasonable request.

## Abbreviations

3D: Three-Dimensional
ASD: Atrial Septal Defect
ECG: Electrocardiogram
IVC: Inferior Vena Cava
LA: Left Atrium
LV: Left Ventricle/cular
mPAP: Mean Pulmonary Artery Pressure
MRI: Magnetic Resonance Imaging
NYHA: New York Heart Association
PASP: Pulmonary Artery Systolic Pressure
PCWP: Pulmonary Capillary Wedge Pressure
PVR: Pulmonary Vascular Resistance
Qp: Qs:Pulmonary-Systemic Flow Ratio
RV: Right Ventricle/cular
TEE: Trans-Esophageal Echocardiography
TTE: Transthoracic Echocardiography
